# On the origins of American Criollo pigs: A common genetic background with a lasting Iberian signature

**DOI:** 10.1371/journal.pone.0251879

**Published:** 2021-05-20

**Authors:** Maria Antonia Revidatti, Luis T. Gama, Inmaculada Martin Burriel, Oscar Cortés Gardyn, Juan Sebastian Cappello Villada, María Inés Carolino, Francisco Javier Cañón, Catarina Ginja, Philip Sponenberg, Antonio P. Vicente, Pilar Zaragoza, Juan Vicente Delgado, Amparo Martínez, the BioPig Consortium

**Affiliations:** 1 Departamento de Producción Animal, Facultad de Ciencias Veterinarias, Universidad Nacional del Nordeste, Corrientes, Argentina; 2 Centre for Interdisciplinary Research for Animal Health, Faculdade de Medicina Veterinaria, Universidade de Lisboa, Lisbon, Portugal; 3 Laboratorio de Genética Bioquímica, Facultad de Veterinaria, Universidad de Zaragoza, Zaragoza, Spain; 4 Departamento de Producción Animal, Facultad de Veterinaria, Madrid, Spain; 5 Instituto Nacional Investigação Agrária e Veterinária, Vale de Santarém, Portugal; 6 Centro de Investigação em Biodiversidade e Recursos Genéticos, Universidade do Porto, Campus Agrário de Vairão, Vairão, Portugal; 7 Virginia-Maryland Regional College of Veterinary Medicine, Virginia Tech, Blacksburg, Virginia, United States of America; 8 Escola Superior Agrária, Instituto Politécnico de Santarém, Santarém, Portugal; 9 Departamento de Genética, Campus de Excelencia Internacional Agroalimentario, Universidad de Córdoba, Córdoba, Spain; National Cheng Kung University, TAIWAN

## Abstract

American Criollo pigs are thought to descend mainly from those imported from the Iberian Peninsula starting in the late 15th century. Criollo pigs subsequently expanded throughout the Americas, adapting to very diverse environments, and possibly receiving influences from other origins. With the intensification of agriculture in the mid-20th century, cosmopolitan breeds largely replaced Criollo pigs, and the few remaining are mostly maintained by rural communities in marginal areas where they still play an important socio-economic and cultural role. In this study, we used 24 microsatellite markers in samples from 1715 pigs representing 46 breeds with worldwide distribution, including 17 American Criollo breeds, with the major focus of investigating their genetic diversity, structure and breed relationships. We also included representatives of the Iberian, Local British, Hungarian, Chinese and Commercial breeds, as well as Wild Boar, in order to investigate their possible influence in the genetic composition of Criollos. Our results show that, when compared with the other breeds, Criollo pigs present higher levels of genetic diversity, both in terms of allelic diversity and expected heterozygosity. The various analyses indicate that breed differentiation overall explains nearly 21% of the total genetic diversity. Criollo breeds showed their own identity and shared a common genetic background, tending to cluster together in various analyses, even though they differ from each other. A close relationship of Criollos with Iberian breeds was revealed by all the different analyses, and the contribution of Iberian breeds, particularly of the Celtic breeds, is still present in various Criollo breeds. No influence of Chinese breeds was detected on Criollos, but a few were influenced by Commercial breeds or by wild pigs. Our results confirm the uniqueness of American Criollo pigs and the role that Iberian breeds have played in their development.

## Introduction

The first domestic swine arriving in the American continent came from the Canary Islands, in the second voyage of Columbus in 1493 [1]. Over a period of expansion and adaptation to local conditions, pigs arriving from the Iberian Peninsula reproduced in large numbers and rapidly expanded throughout the continent [2–4]. As happened with other livestock species in the Americas, the descendants of pigs of Iberian ancestry are known as “Creole” or “Criollos”[5, 6], and they include a wide variety of populations with different phenotypic characteristics and adapted to an enormous variety of environments and production systems, spreading from the United States to Argentina, and from tropical climates to nearly desert conditions [7].

The intricacies of the American colonization process and the result of nearly 500 years of adaptation to distinct environmental conditions have contributed to the highly heterogeneous genetic pool currently represented by Criollo pigs. In addition, various pig breeds from different origins, including China, have arrived in America over the years and may have been mixed with local populations, adding to the complexity of the genetic structure of Criollo pig populations [8].

The intensification of pig production throughout the world in the second half of the 20th century has threatened many local populations, and Criollo pigs were led to near extinction. The few remaining populations currently existing are widely spread, often with very small census. Criollo pigs are now mostly maintained by rural communities in marginal areas, where village pigs are usually kept in a commensal relationship where they play an important role in sustainable economies by using local feed resources and converting domestic waste into animal protein [9].

In addition to their social and historical importance, the elements of isolation and adaptation to extremely different environmental conditions that Criollo pigs experienced make them a highly valuable biological resource. They have had to adjust to very diverse climates, feed sources, health constraints, management systems, and disease threats. Their adjustments provide for the possibility of gaining a better understanding of the genetic background underlying the various mechanisms of adaptation that these populations have undergone [10].

Studying the genetic history of Criollo pigs is essential for a better understanding of the importance of livestock throughout the period of discovery and colonization of the Americas, and how the introduction of breeds of other origins may reflect changes in food habits and agricultural practices over time. Furthermore, understanding the genetic diversity and structure of Criollo pigs is crucial for a better awareness of their importance and for the establishment of management programs aimed at their conservation.

The genetic diversity and relationships of some Criollo pig breeds [11] as well as the analysis of their conservation priorities [10] have been analyzed with microsatellite markers, and their origins have been investigated with single nucleotide polymorphisms [8]. In our study we used a comprehensive genetic sampling of Criollo and other pig breeds of cosmopolitan importance. Our analyses were based on a broad representation of worldwide pig populations, including 1715 DNA samples representing 17 Criollo and 29 breeds/populations of other origins, including 15 Iberian, 5 Commercial, 4 Wild Boar, 3 Local British, 1 Chinese and 1 Hungarian breed. We used a set of widely used neutral genetic markers to assess the genetic diversity, relationships and possible admixture with other breeds in Criollo pig populations. Furthermore, we also investigated genetic bottlenecks that may have occurred in the various populations that could have affected their genetic variability.

## Materials and methods

### Pig breeds

**Fig 1:**
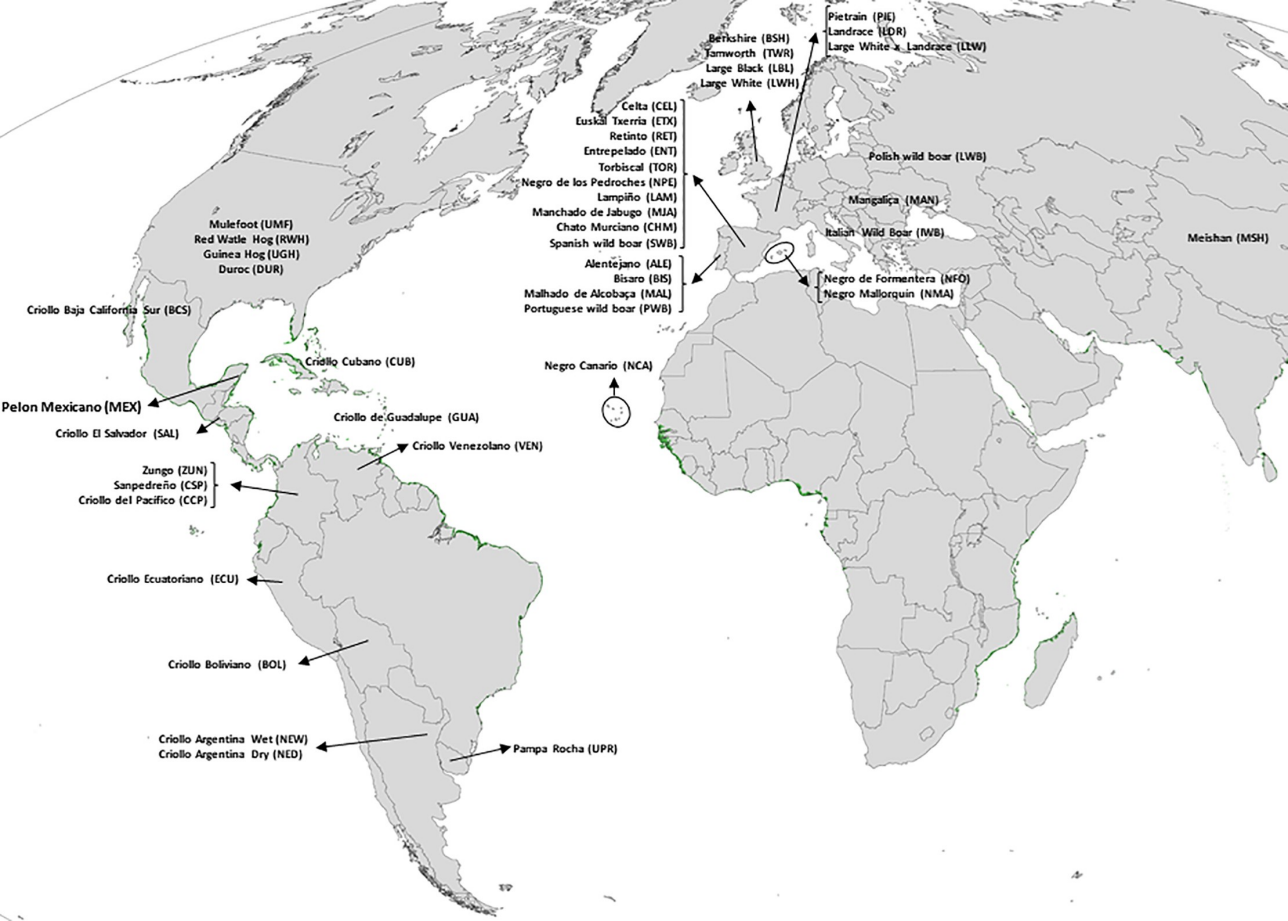
Geographical distribution of the 46 pig populations/breeds analyzed. Map source: source: https://eoimages.gsfc.nasa.gov/images/imagerecords/47000/47427/global_tm5_mangroves_lrg.png.

A set of 24 microsatellite markers was used to analyze samples of 46 populations/breeds belonging to different countries/regions, as represented in Fig 1. In order to clarify the genetic influences underlying the development of Criollo pigs, the analysed breeds were classified in the following groups: **Criollos (CRI):** including animals from five populations from North America: Mulefoot (UMF), Red Wattle Hog (RWH), Guinea Hog (UGH) from the United States and Pelón Mexicano (MEX) and Baja California (BCS) from Mexico; one breed from Central America: Criollo Salvadoreño (SAL) from El Salvador; two breeds representing the Caribbean Islands: Criollo Cubano (CUB) from Cuba and Criollo de Guadalupe (GUA) from Guadeloupe Island and nine breeds from South America: Criollo Venezolano (VEN) from Venezuela; Zungo (ZUN), San Pedreño (CSP), and Criollo del Pacífico (CCP) from Colombia; Criollo Ecuatoriano (ECU) from Ecuador; Criollo Boliviano (BOL) from Bolivia; Pampa Rocha (UPR) from Uruguay; and Criollos from either Northeast Argentina Wet (NEW) and Dry (NED) regions; **Iberian Peninsula breeds (IBE):** including Retinto (RET), Entrepelado (ENT), Torbiscal (TOR), Negro de los Pedroches (NPE), Lampiño (LAM), Manchado de Jabugo (MJA), Chato Murciano (CHM), Negro Canario (NCA), Negro de Formentera (NFO), Negro Mallorquín (NMA), Euskal Txerria (ETX), and Celta (CEL), from Spain; Alentejano (ALE), Bisaro (BIS) and Malhado de Alcobaça (MAL) from Portugal; **Local British breeds (LBR):** composed by Berkshire (BSH), Tamworth (TWD) and Large Black (LBL) from UK; **Commercial breeds (COM)**: consisting of five cosmopolitan breeds, Duroc (DUR), Pietrain (PIE), Large White (LWH), Landrace (LDR), and the commercial crossbred population Large White x Landrace (LLW); the **Hungarian** Mangaliça (MAN) breed; the **Chinese** Meishan (MSH) breed; **Wild boar (WBO) populations**: composed by wild boar samples obtained in Portugal (PWB), Spain (SWB), Poland (LWB) and Italy (IWB).

The biological samples for genotyping were obtained in the framework of the CONBIAND network, as described by Gama et al. [12] for Iberian Peninsula breeds, Revidatti et al. [11] for Criollo pig breeds and Martinez et al. [13] for Wild Boars from Spain, Portugal and Italy. The sampling and genotyping of Commercial breeds other than PIE were performed by our group, with the procedures described by Gama et al. [12] and Revidatti et al. [11]. The genotypes for Local British breeds, and for PIE, GUA, MAN, LWB and MSH were obtained from the PigBiodiv European Project database [14]. Overall, the total number of pigs sampled was 1715 and the sample size by population varied between 12 and 50, as shown in S1 Table. Biological samples (blood or hair roots) for genotyping were collected by qualified veterinarians during their routine practice, in the framework of official programs aimed at identifying and controlling the health and confirming parentage of the populations/breeds included in the current work.

### Microsatellite markers and genotyping

Individual genotyping has been previously reported by Gama et al. [12] for Iberian Peninsula breeds, by Revidatti et al. [11] for Criollo pigs and by Cortes et al. [10] for British local and Commercial populations. The following 24 microsatellite markers recommended for genetic diversity studies by the International Society for Animal Genetics/Food and Agriculture Organization of the United Nations [15] were used for the analyzes: *IGF1*, *S0002*, *S0005*, *S0026*, *S0068*, *S0090*, *S0101*, *S0155*, *S0178*, *S0215*, *S0225*, *S0226*, *S0227*, *S0228*, *S0355*, *S0386*, *SW024*, *SW072*, *SW240*, *SW632*, *SW857*, *SW911*, *SW936* and *SW951*.

### Data analysis

The data analyzes were performed using individual samples, considering the breeds/populations and groups described above. Total number of alleles (tA), the mean number of alleles (MNA) and the unbiased expected (H_e_) and observed (H_o_) heterozygosities per breed and group were calculated with the MICROSATELLITE Toolkit software for Excel [16]. In the analyses of breed groups, all animals of the same group were analyzed together, regardless of their breed of origin.

Allelic richness (Rt), which corresponds to the corrected mean number of alleles per locus for each breed/group accounting for sample size, was calculated assuming a minimum of 9 animals per breed and 25 animals per group and was obtained using FSTAT v. 2.9.3 [17]. The effective number of alleles (Ae) was calculated using POPGENE v. 1.31 [18].

The GENEPOP v. 4.7 [19] program was used to test for deviations from Hardy-Weinberg Equilibrium (HWE) per breed, using a Markov chain method to estimate the p-values. Fisher’s method was applied to calculate the significance of the HWE probabilities across loci. FSTAT v. 2.9.3 [17] was used to estimate the F statistics per group and breed, according to Weir & Cockerham (1984) [20], and p-values were obtained based on 1000 randomizations.

A hierarchical analysis of variance was performed to analyze the partition of the total genetic variance into components due to group, interpopulation variability and interindividual differences. Computations were carried out using the AMOVA (Analysis of Molecular Variance) module of ARLEQUIN 3.5 [21].

#### Genetic distances and factorial correspondence analysis

Genetic distances among breeds and breed-groups were estimated by theta values [20] with GENETIX v. 4.04 software [22], such that all animals of the same breed-group were combined, ignoring their breed of origin. The Nei D_A_ genetic distances were estimated in POPULATIONS v. 1.2.28 [23]. The Neighbor-Net method [24] as implemented in SPLITSTREE4 v. 4.14.4 software [25] was used to compute a network based on Nei distances to graphically represent breed relationships and admixture. The Factorial Correspondence Analysis (FCA) considering data for populations and groups of populations were performed using the GENETIX v. 4.04 software [22].

#### Genetic structure

The Bayesian model-based method developed by Pritchard et al. [26] and implemented in the STRUCTURE v. 2.3.4 software was used to investigate population structure and define clusters of individuals on the basis of multi-locus genotypes for the 24 microsatellite markers. The number of assumed populations (K) varied between 1 and 40. For each K, 10 independent runs were performed with a burn-in period of 10^5^ iterations and Monte Carlo Markov Chain (MCMC) length of 5x 10^5^ iterations under an admixture and correlated allele frequencies model. The average and standard deviation of the logarithm of the likelihood function [L(K)] of the data were estimated across 10 runs for each K value. The most probable number of population clusters was determined by plotting L(K) and also with the distribution of ΔK [27] calculated in the website STRUCTURE HARVESTER v. 0.6.94 [28]. The cluster outputs of the 10 independent runs for each value of k were combined with CLUMPP v. 1.1.2 [29], which was used to select the best solution. After assessing the most likely number of underlying populations, the results were graphically displayed with Excel.

#### Effective population size and demography

Effective population size was estimated with NeESTIMATOR V2 [30] using the linkage disequilibrium-based method that tests for nonrandom association among alleles at different loci that occur when effective size (Ne) is low and genetic drift influences allelic frequencies [31]. The lowest allele frequency was defined at 0.05 to exclude single copy alleles, as rare alleles tend to bias linkage disequilibrium estimates upward [32].

The occurrence of genetic bottlenecks was investigated by two different approaches, (1) heterozygosity excess and (2) Garza-Williamson index (GW). In (1), the BOTTLENECK software was used to test for excess genetic diversity relative to that expected under mutation-drift equilibrium [33], as the allele diversity of a population is expected to decrease faster than heterozygosity during a bottleneck. The distribution of heterozygosity from the observed number of alleles (m), given the sample size (n) under the assumption of mutation-drift, is obtained by a coalescent process of n genes under a Two-Phase Model (TPM) (95% stepwise mutation model with 5% multistep mutations and a variance among multiple state of 12) using the Wilcoxon test, that is considered the optimal parameter for microsatellite data [34]. The GW statistic in (2) was estimated as proposed by Garza and Williamson [35], using ARLEQUIN software v. 3.11 [21], and the following formula:

$$GW=\frac{m}{R+1}$$

where *m* is the number of alleles in a given locus in a population sample and *R* is the allelic range. The GW statistic is expected to be very small in populations having been through bottlenecks and close to one in stationary population.

## Results

### Genetic diversity

The diversity estimates observed for the various groups are in Table 1. The means for tA, MNA, Ae, Rt show a similar behaviour, with the lowest values found in the MAN breed, with means of 76, 3.17 ± 1.27 1.99 ± 0.74nd 3.16 ± 1.21 alleles, respectively. On the other hand, the highest means for allelic diversity were observed in CRI, with mean values of 332, 13.83 ± 5.00, 4.64 ± 2.87 and 7.98 ± 2.09 alleles for tA, MNA, Ae and Rt, respectively. It is interesting to notice that the mean values for allelic diversity observed in the IBE group are the closest to those found in CRI. The MAN breed also showed the lowest estimates for H_e_ and H_o_ (0.424 ± 0.050 and 0.347 ± 0.020, respectively) and the highest values were observed in COM (0.708 ± 0.030 and 0.581 ± 0.008, respectively). The WBO had levels of genetic diversity slightly lower than those observed in CRI and IBE. The deficit in heterozygosity computed within each breed-group was significant in all breed-groups (p<0.001) and resulted in an *f* estimate with the highest values found in IBE (0.271), followed by LBR (0.236) and CRI breeds (0.194). The majority of the breed-groups were composed by more than one breed, therefore the *f* estimate should reflect the subdivision of populations forming the groups, in addition to their accumulated inbreeding. However, in the MSH and MAN groups only one breed is being considered, thus the estimated *f* should reflect mostly inbreeding rather than breed substructure.

**Table 1:** Diversity estimates for groups of populations.

*Population groups*	*Acronym*	*n*	*tA*	*MNA*	*Rt*	*Ae*	*H*_*e*_	*H*_*o*_	*f*
				*(SD)*	*(SD)*	*(SD)*	*(SD)*	*(SD)*	
Criollo^1^	CRI	605	**332**	**13.83**	**7.98**	**4.64**	0.702	0.566	0.194***
				**-5**	**-2.09**	**-2.87**	-0.036	-0.004	
Iberian peninsula^2^	IBE	622	281	11.71	6.88	3.9	0.663	0.484	0.271***
				-4.72	-2.22	-1.81	-0.043	-0.004	
Local British^3^	LBR	135	144	6	4.87	3.27	0.64	0.489	0.236***
				-1.98	-1.47	-1.29	-0.033	-0.009	
Commercial^4^	COM	170	200	8.33	6.04	4.08	**0.708**	**0.581**	0.180***
				-3.38	-1.72	-1.7	**-0.03**	**-0.008**	
Mangaliça	MAN	25	**76**	**3.17**	**3.16**	1.99	**0.424**	**0.347**	0.185***
				**-1.27**	**-1.21**	-0.74	**-0.05**	**-0.02**	
Meishan	MSH	45	105	4.38	4.37	**2.66**	0.584	0.522	0.108***
				-1.47	-1.18	**-0.99**	-0.033	-0.016	
Wild boar^5^	WBO	113	216	9	6.23	4.08	0.648	0.549	0.152***
				-4.18	-2.53	-2.69	-0.047	-0.01	

Group, corresponding acronym, sample size (n) and diversity estimates: total number of alleles observed in each group (tA), mean number of alleles per locus (MNA), allelic richness (R_t_), effective number of alleles (Ae) expected (H_e_) and observed (H_o_) heterozygosities, heterozygosity deficit (*f*) and its significance (*** *p*<0.001). The maximum and minimum values are shown in bold and standard deviations (SD) are in parentheses.

^**1**^**Criollo:** Mulefoot, Red Wattle Hog, Guinea Hog, Criollo Baja California, Pelón Mexicano, Criollo Salvadoreño, Criollo Cubano, Criollo de Guadalupe, Criollo Venezolano, Zungo, San Pedreño, Criollo del Pacifico, Criollo Ecuatoriano, Criollo Boliviano, Pampa Rocha, Criollo Argentina Wet, Criollo Argentina Dry

^**2**^**Iberian peninsula:** Retinto, Entrepelado, Torbiscal, Negro de los Pedroches, Lampiño, Manchado de Jabugo, Chato Murciano, Negro Canario, Negro de Formentera, Negro Mallorquín, Euskal Txerria, Celta, Alentejano, Bisaro, Malhado de Alcobaça

^3^**Local British:** Berkshire, Tamworth, Large Black

^4^**Commercial**: Duroc, Pietrain, Large White, Landrace, Large White x Landrace

^5^**Wild boar:** Portuguese wild boar, Spanish wild boar, Polish wild boar, Italian wild boar.

When genetic diversity was assessed by breed within group (S1 Table), the highest means for allelic and genetic diversity in the CRI group were found in the ECU and the lowest in the UMF. On the other hand, for the IBE group, the highest and lowest estimates of allelic and genetic diversity were found in ALE and MJA, respectively. Across the 46 breeds analysed, the lowest levels of genetic diversity were found in BSH and UMF, while the highest genetic diversity was detected in ECU followed by NEW. Differences between the expected and observed heterozygosities in the various breeds analysed indicated a statistically significant *f* (p<0.05) in 28 populations, with the highest *f* estimate of 0.285 observed in the MEX population. All breeds except CSP, UPR and BSH displayed departure from Hardy Weinberg Equilibrium at least in one locus (p<0.05).

The average F-statistics and their standard deviation were *f* = 0.073±0.007, *Ө* = 0.210±0.006 and *F* = 0.267±0.009. These results reveal that the levels of breed differentiation were considerable, with multilocus *Ө* values indicating that approximately 21% of the total genetic variation corresponded to differences between breeds, while the remaining 79% were attributed to differences among individuals within breeds.

Partitioning of genetic variability among the different sources of variation (AMOVA test) indicated that nearly 4.4% of the total genetic variability was explained by the assumed breed-groups, whereas the differences among populations/breeds within groups represented about 17% of the total variability (S2 Table). The corresponding fixation indexes in AMOVA confirmed that the overall differentiation between breeds (f_ST_ = 0.215) is mostly due to differences among breeds within the same region (f_SC_ = 0.178) rather than differences between regions (f_CT_ = 0.044).

### Genetic distances

Genetic distances among breed-group pairs, estimated by pairwise theta values, indicated that the MAN and MSH had the largest distances relative to the other groups. The smallest distance was found between the CRI and IBE groups (*Ө* = 0.021), supporting their much closer relationship. The CRI then had distances of about 0.04 with the COM group and about 0.06 with the LBR and WBO. The estimated Nei distances pointed to the same relationships, i.e., a much closer relationship of CRI with IBE (D_A_ = 0.061), while the distances with COM, WBO and LBR were increasingly larger. It should also be noticed that, for the CRI group, the distance with the MSH breed estimated by both methods was much larger than for any of the other breed-groups (Table 2).

**Table 2:** Genetic distances estimated between population groups^1^, with Weir and Cockerham theta distance shown above the diagonal and Nei’s distance below the diagonal.

	CRI	IBE	LBR	COM	MAN	MSH	WBO
CRI	-	0.021	0.064	0.042	0.164	0.297	0.058
IBE	0.061	-	0.086	0.059	0.168	0.322	0.051
LBR	0.160	0.195	-	0.078	0.229	0.314	0.138
COM	0.107	0.141	0.176	-	0.190	0.273	0.106
MAN	0.300	0.285	0.361	0.360	-	0.438	0.183
MSH	0.628	0.670	0.637	0.605	0.755	-	0.343
WBO	0.136	0.113	0.286	0.261	0.289	0.729	-

^1^ See Table 1 for specification of breeds/populations belonging to each group.

**Fig 2:**
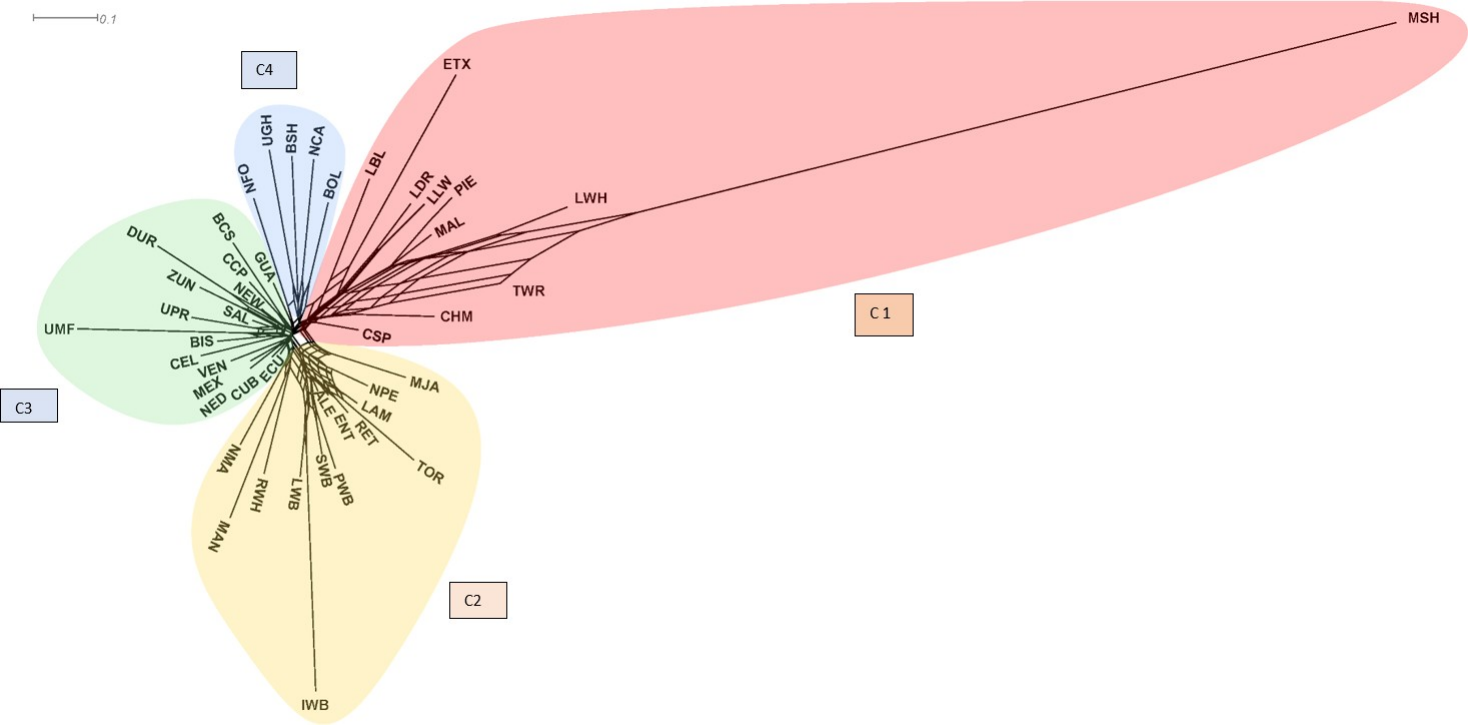
Neighbor-net dendrogram constructed from Nei’s genetic distances among 46 pig populations (See Fig 1 for definition of breed acronyms).

The Neighbour-net built with the D_A_ genetic distance of Nei (Fig 2) supports the existence of four major clusters. The first cluster (C1 in Fig 2) included MSH, all commercial populations, except Duroc, two of the three British local breeds (TWR and LBL), three Iberian Peninsula populations (MAL, CHM, ETX) and only one Criollo population belonging to Colombia (CSP). The second Cluster (C2) grouped the populations of the Iberian Peninsula that belong to the Mediterranean group plus the four wild boar populations and the MAN; however, no CRI population was included in this cluster. The C3 cluster grouped most of the Criollo populations together with CEL, BIS, and DUR, demonstrating a remarkable proximity among CRI and suggesting the influence of the two Iberian breeds and the possibility that the Duroc actually belongs to the CRI group. The last cluster (C4) grouped two CRI populations (BOL and UGH) as well as two of the IBE breeds (NCA and NFO) and the local British BSH.

### Genetic structure

**Fig 3:**
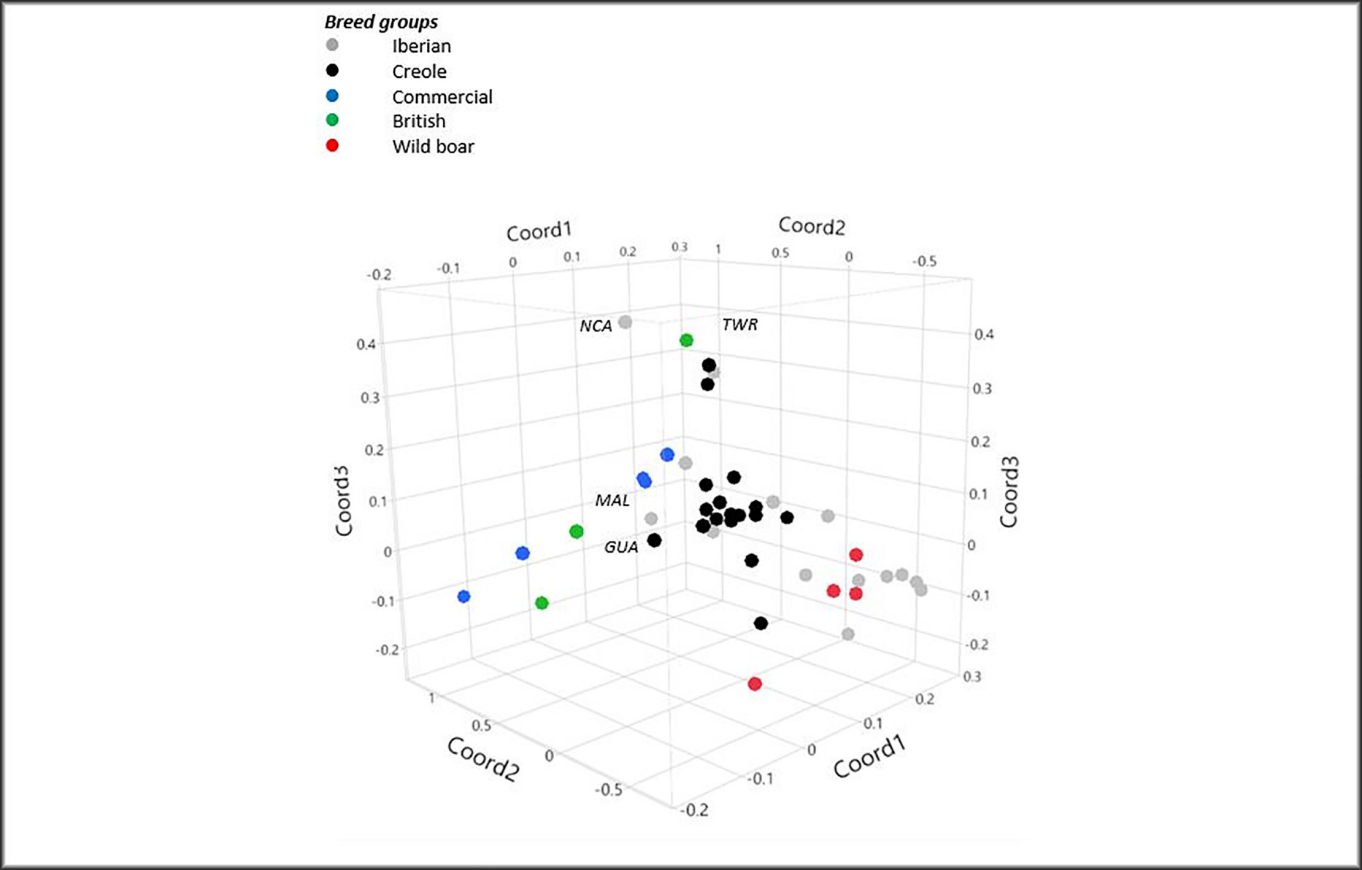
Graphical representation of the three first axes from the factorial correspondence analysis of the 46 pig populations, where coordinates 1, 2 and 3 explained, respectively, 8.5, 5.9 and 5.5% of the total variance. Breeds deviating from their group are indicated.

The genetic structure of the 46 analysed populations was further assessed by Factorial Correspondence Analysis, where the first three components accounted for 19.9% of the total variability. The three-dimensional plot of breed coordinates defined by the first three major axes is in Fig 3, excluding in this representation the MSH, MAN and ETX breeds, as these were outliers in the distribution. In the FCA analysis, the distribution of breeds according to axes 1 and 2 clearly resulted in the separation of the IBE, CRI and WBO populations on one side, and the remaining breeds on the other side. There was no clear separation between the IBE and CRI populations, which would lend support to the evidence of an Iberian influence on the majority of Criollo populations. A few Iberian breeds where the possible influence of exotic germplasm has been suggested, such as MAL and NCA, tend to separate from the major cluster of Iberian breeds, and the same occurred with the GUA.

Bayesian clustering methods allow for the assignment of individuals to groups based on their genetic similarity and provide information on the number of ancestral populations underlying the observed genetic diversity. The results of these analyses using the STRUCTURE software [26] indicated that the most likely number of ancestral populations, estimated according to Evanno et al. [27], is K = 29, as concluded from the modal distribution of ΔK (S1 Fig).

**Fig 4:**
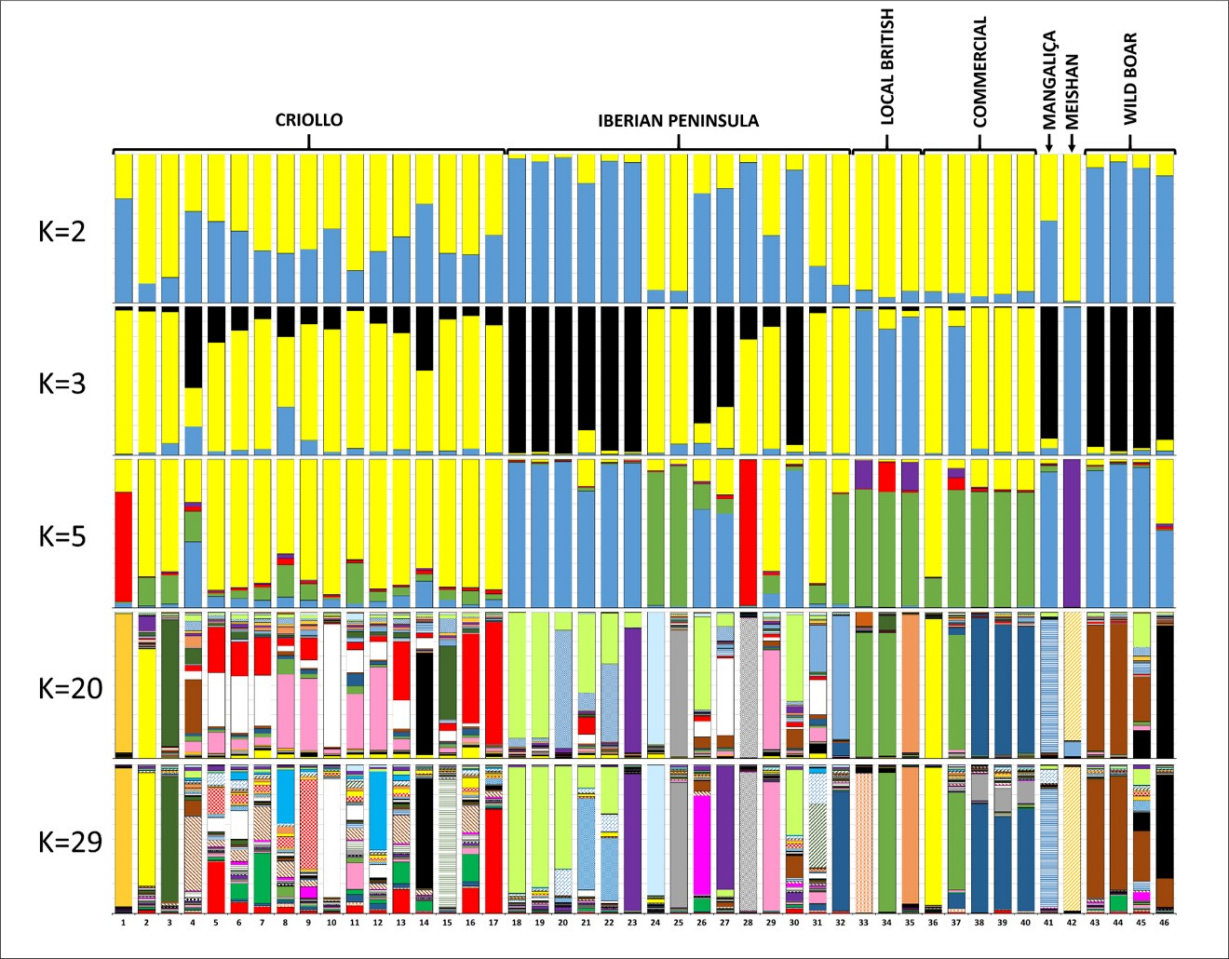
Population structure of 46 pig population based on 24 microsatellite loci using the STRUCTURE software. Each breed is represented by a single vertical bar divided into K colours, where K is the number of assumed ancestral clusters, which is graphically represented for K = 2, 3, 5, 20 and 29.

The results of the Bayesian cluster analyses are summarized in Fig 4, for values of K = 2, 3, 5, 20 and 29. When only 2 ancestral populations were assumed (K = 2), WBO and nine Mediterranean breeds that compose the Iberian Peninsula group, together with the Basque breed ETX, formed the first cluster. The other cluster was formed by Commercial and Local British breeds, the MSH, a few Iberian breeds of the Celtic group (CEL, BIS, MAL), plus the CHM and NCA. The Criollo breeds as well as the MAN generally displayed mixed contributions of the two clusters.

When a number of ancestral populations of K = 3 is assumed, the WBO and nine of the IBE breeds (consisting mostly of Mediterranean breeds) clustered together and MAN was added to this group. Another group was represented by British local breeds, PIE and MSH. The third cluster grouped the IBE breeds of the Celtic group, the CHM, NCA and ETX, and nearly all the Criollo populations.

At K = 5, the MSH separated from the other groups, while the UMF and ETX clustered together. The CRI group (with the exception of UMF and BCS) was mostly homogeneous and clustered with the CEL and BIS from the Iberian Peninsula, and the DUR from the COM group.

The COM breeds grouped with CHM, NCA and the local British breeds. The Iberian breeds of the Mediterranean group remained in the same cluster with the WBO group.

At K = 20, breeds in the Criollo group generally showed different ancestry relative to each other and, with very few exceptions, no influence could be detected from the MSH, commercial or British local breeds. Overall, Criollo breeds were either essentially distinct from all the other breed groups or showed signs of an Iberian influence. For example, the UMF was well isolated from the remaining breeds, while the UGH and UPR grouped in the same cluster but no relationship was detected with other breed groups. Moreover, RWH and DUR clearly showed signs of admixture, and some Criollo breeds such as BCS and BOL showed influence of WBO. The NEW and NED were well differentiated from most of the other breeds, but shared a common admixture pattern with MEX, SAL, CUB and ECU. On the other hand, the MEX, SAL and CUB also appeared to share a common genetic background with the Colombian breeds (ZUN, CSP and CCP) and the ECU, as well as with the Iberian breeds of the Celtic group, which may reflect an influence of breeds such as BIS and NMA on Criollos. The influence of the CEL breed could also be detected in GUA, VEN, CSP and CCP, confirming the influence of Iberian Celtic breeds in various Criollo populations.

Overall, at K = 20 the MAN and MSH breeds were well isolated from all the other breeds, and the commercial breeds LWH, LDR and LLW remained closely grouped. In local British breeds, the LBL separated from the BSH and TWR, which shared a common ancestry. In the wild boar, some separation occurred according to geographical origin, with the Italian group clearly isolated and the Polish group showing some signs of substructure. Among the Iberian pig breeds, the Mediterranean group of breeds clustered together, with some separation of the TOR and LAM breeds. Yet, most of the other Iberian breeds were well isolated and showed influence of a single ancestral population, possibly with the exception of BIS, which had signs of mixed ancestry.

At K = 29, the clusters for individual breeds and groups were essentially the same described for K = 20. Again, the majority of the Criollo breeds had a mixed ancestry, and in most cases, they shared a common blended ancestry with each other. Nevertheless, a few of them were well isolated (for example, UMF), were closely related (UGH and UPR), or showed a clear influence from another breed (RWH from DUR and BOL from WBO).

### Effective population size and demography

The effective population size (Ne), estimated from linkage disequilibrium, differed considerably among the various breeds analyzed (S3 Table). Overall, the Ne estimate ranged from 2.1 to 63.9 in IBE breeds, and it was particularly low for some breeds which are known to have undergone bottleneck events over the last decades (for example, NPE, MJA, CHM, NFO and BIS). The estimated Ne was generally above 20 for most CRI breeds, but it was below 10 for the UGH and CSP, and above 50 for the BCS, SAL, CUB, ZUN, CPA and ECU. The Ne estimates in BRI and COM groups ranged from 13.4 to 78.5.

When Bottleneck software was used (S3 Table), heterozygosity excess could be detected (p<0.05) in three IBE population (CEL, MAL and BIS), two British (BSH and TAM), two COM (DUR and LDR) and the MSH breed. On the other hand, CRI populations did not show any signs of bottleneck under the TPM mutation model. In general, GW index values higher than 0.68 are representative of populations without signals of recent reduction in size [35]. While in our study the GW index ranged from 0.5 to 0.76, it is remarkable that GW values above 0.68 could only be detected in four CRI populations (CUB, ECU, NED and NEW).

## Discussion

It is well established that domestic swine did not exist in the American continent until 1493, when the first pigs and other livestock were brought from the Canary Islands in the second journey of Columbus. The process of recruitment, transportation and expansion of this first group of pigs brought to the New World is documented in the writings of the Dominican monk Bartolomé de las Casas, a narrator who described in detail the early years of discovery and colonization of the American continent in his treaty “Historia de las Indias” [36]. In this work, friar Bartolomé de las Casas provides details on the arrival of pigs in America when he describes “[some of Columbus’s sailors] bought eight sows… And these eight sows multiplied and originated all the pigs that have existed and now exist in these Indies, which are an infinite number”. While this statement should not be interpreted literally, it makes clear that the initial expansion of pigs in the Americas was explosive, and essentially resulted from a narrow original genetic foundation.

In the newly discovered territories, pork was often the only familiar meat available to the first colonists, and lard was an essential ingredient for several human activities [37]. Of the various livestock species introduced from the Iberian Peninsula, the pig adapted most quickly to the new environment and, given its high reproductive rate, it rapidly expanded throughout the vast territories of the New World. The number of pigs increased so dramatically that, 50 years after their arrival to the Americas, “swine herds were to be found wherever the Spanish settled or even touched, and the same was true in the Portuguese areas” [2].

As for other livestock species, these pigs of colonial descent became known as Criollos, and they expanded through every region of North and South America, adapting to a vast array of environments, including a wide range of conditions in terms of altitude, temperature, rainfall, etc. In addition to their primary role of providing food for local populations, Criollo pigs also gained importance by their ability to utilize native feed resources and convert domestic waste into pork, and expeditions often brought supplies of preserved salt-pork, which was essential for their maintenance for long periods of travel [37].

Over the period of more than five centuries since their introduction in the Americas, Criollo pigs have undergone a process of fragmentation into various sub-populations. These have endured selection for adaptation to different environmental constraints, and genetic drift and bottlenecks have likely occurred over time. It is also possible that, throughout this period, exotic germplasm of European or Asian origin have been introduced in some Criollo populations [38, 39], including direct importation of animals from the United Kingdom, where some of the breeds already had the influence of Chinese pigs [40]. The second half of the 20th century was the turning point, when the intensification of agriculture led to the extensive use of a few cosmopolitan pig breeds all over the world, including the Americas. Since that time, many Criollo pig populations have been admixed or abandoned, and the ones that still remain have been maintained mostly by indigenous populations and small farmers in marginal production systems, for whom pigs remain an important economic resource and a key element of local culture and traditions [9].

Some research on Criollo pigs has relied on mitochondrial DNA sequences and Y-chromosome markers to elucidate their origins [39, 41, 42]. Also, genetic diversity was examined in some populations using a high-density panel of SNPs [8]. Studies with microsatellites markers have also been reported on some Criollo populations, but were limited to a relatively small number of breeds, or restricted to animals from particular countries or regions [12, 43–54].

The possible influence that other pig breeds may have had on the current genetic structure of American Criollo populations has not been thoroughly investigated so far. In this work, we used microsatellite markers to assess genetic diversity, genetic relationships and the possible influences that pig breeds from Spain and Portugal, as well as other European breeds (including wild boars), commercial breeds and Chinese Meishan, may have had in the development of Criollo pig populations. Our study provides important information regarding the genetic diversity and structure of Criollo pig breeds, and also a new perception of the relationships among them and of their possible ancestral origins.

Our results clearly show that Criollo pigs present high levels of genetic diversity, both in terms of allelic variation and expected heterozygosity. Indeed, the genetic diversity of Criollos is higher than that found in any of the other groups studied. This may reflect the history of Criollo pigs, including population fragmentation followed by genetic drift in the past, as well as the consequences of possible admixture with imported pig breeds. On the other hand, this high genetic diversity may also be a consequence of adaptation to very diverse environmental conditions of the various Criollo populations, which would lead to population divergence in case the microsatellite markers are not completely neutral or are linked to genomic regions under selection [55]. In a recent study with Criollo cattle populations, Ginja et al. [56] also found higher levels of genetic diversity in Criollos when compared with Iberian, Continental, British, African and Indicus cattle, and Cortés et al. [57] also report higher levels of genetic diversity in Criollo horses when compared with breeds from other origins. However, in other studies with Criollo populations, Sevane et al. [58] with goats and Jordana et al. [59] with donkeys, did not find higher levels of genetic diversity in Criollos when compared to other breeds. This could certainly reflect the different historical patterns of development and dispersal of the various domestic species in the American continent [2, 60], which have shaped their current diversity and structure.

At the breed level, large differences were observed, not only among the 46 breeds with a worldwide distribution included in our analyses, but also among the 17 Criollo breeds represented. The highest levels of genetic diversity in Criollo pigs were found in ECU from Ecuador and NEW from Argentina, and these are also the breeds which, in the analyses with STRUCTURE, reveal some of the more diversified contributions of ancestral populations. On the other hand, the Criollo breeds with the lowest levels of genetic diversity were the UMF and RWH, both from the United States. In the analyses with STRUCTURE, the UMF seems to be very homogeneous and to have a unique ancestral contribution, while the RWH is closely related with the Duroc; thus, the low genetic diversity observed in these two populations probably reflects past population decline or a possible founder effect.

The majority of the Criollo breeds shows a significant deficit in heterozygosity. This likely reflects their reduced census sizes or possibly the existence of substructure in some populations, as a consequence of fragmentation and genetic isolation. Still, the analyses carried out to assess past effective population size did not reveal, for the majority of Criollos, the occurrence of census reduction nor the existence of strong bottlenecks in the past. On the contrary, various Iberian breeds have indications of strong reductions in effective population size, which is compatible with their recent history [61].

For selectively neutral loci such as microsatellites, a reduction in allele number and heterozygosity are anticipated when a bottleneck occurs and the effective population size is reduced. However, they are expected to occur at different rates [33]. Under these circumstances, allelic diversity is reduced faster than heterozygosity and the observed number of alleles is less than what would be expected if the mutation-drift equilibrium is assumed. Therefore, a significant number of loci with allele deficiency might be the consequence of a recent bottleneck [62]. The GW-ratio test, computed based on the number of microsatellite alleles considering the range in allelic size, is expected to be smaller in populations experiencing a bottleneck than in populations at equilibrium [35]. Also, the GW-ratio changes more slowly after a bottleneck because new alleles from mutations do not necessarily increase it and heterozygosity excess is expected to regain mutation-drift equilibrium more rapidly [34]. Thus, a bottleneck in the more distant past is inferred when the GW-ratio but not the heterozygosity test are significant and conversely a more recent decline in population size is inferred to have occurred when heterozygosity but not GW-ratio is significantly different from expectations [63–66].

Assuming a critical value for the GW-test of 0.68, as derived from putatively stable populations [35], in our analyses all the Iberian Peninsula, British and Commercial breeds analyzed achieved GW-ratios lower than this critical value, indicating that bottlenecks could be inferred to have occurred in those populations. It is well known that native pig breeds in the Iberian Peninsula have experienced a genetic bottleneck mainly at the mid-20th century, due to intensification of agriculture and African swine fever outbreaks [12]. By the end of the 20th century, an increase in population size of these local Iberian breeds was observed, as a consequence of greater demand for high quality processed products [67]. A similar demographic history could have been experienced by the British breeds, which during the second half of the 20th century diminished their number due to different factors such as intensification of animal production and urban development. Among the Criollo pig breeds, the CUB, ECU, NEW and NED were the breeds with GW-ratios higher than 0.68 and without heterozygosity excess. The population size of the CUB increased significantly since the middle of the 20th century, due to changes of the production systems and this could explain the absence of bottleneck signals in comparison with other Criollo pig breeds, which have since this time decreased their census [68]. The NEW and NED populations from northeast Argentina are semi-feral populations without a systematic management, maintained in a production system that could result in the maintenance of a stable population size over the years [69]. Finally, the ECU had a GW-ratio of 0.76, indicating a high effective number of alleles which is probably a consequence of crossbreeding with other pig populations [7] that have changed the allele distribution in terms of number and range in allele size and could explain the high GW-ratio value. Furthermore, the ECU did not reveal heterozygosity excess, reinforcing the idea of an absence of bottleneck in this population.

In our study, the estimated F-statistics indicated that nearly 21% of the total genetic diversity corresponded to breed differentiation, an estimate which is in line with the AMOVA results that indicate that the most important source of variability was the diversity among populations within groups (about 17%), while the fraction corresponding to the differentiation due to groups was only about 4% of the total variability. The high θ estimate in our study is in agreement with the results reported by Gama et al. [12] for native pig breeds from Iberia and its islands (20%) and other studies with European pig breeds, where θ has ranged from 21% [70] to 27% [71]. However, our estimate is higher than some results reported for Criollo pig populations alone (11%) [11] or for Colombian pig breeds (10%) [72]. The degree of breed differentiation found here is also substantially higher than the between-breed diversity component reported in studies with other livestock species where Criollo breeds were also included, such as cattle [5], donkeys [59], horses [57] and goats [58]. These differences probably reflect the distinct genetic history, reproductive strategies and biological constraints of the various species, with a higher reproductive rate and degree of reproductive isolation and reduced gene flow between breeds in pigs, when compared with other livestock species [73], leading to a sharper breed differentiation.

Our results regarding breed structure and relationships strongly support the idea that Criollo breeds of pigs have their own identity and share a common genetic background, as they tend to cluster together in distance trees and in FCA analyses. However, no clear geographical pattern of distribution of genetic diversity could be identified. The closer relationship of Criollos with Iberian breeds was unquestionable, as revealed by all the different analyses, including Ө, Nei distance tree and FCA, where they are closer to Iberian breeds than to any of the other breed groups. Also clear was the lack of support for a possible contribution of Meishan pigs to Criollos, contrary to some speculations that have been made suggesting that the introduction in America of pigs from China, which probably took place between the 16th and 18th centuries [39, 74, 75], could have left some mark in Criollos. Nevertheless, no evidence of this event was revealed in our study.

It is also interesting to notice that very few Criollos actually showed a close influence from commercial breeds, the major exception being the RWH, which clearly shares a common ancestry with DUR. This likely reflects a Criollo origin to the Duroc breed, which was developed in the United States from local genetic resources in the 1800s. Further evidence of the singular origin of Duroc is provided by its unique position relative to other commercial breeds. Given the expansion that cosmopolitan pig breeds have had all over the world, where they have often had an impact upon and endangered many local breeds [76], it could be anticipated that some Criollo populations would show signs of admixture with COM breeds. This was in fact very seldom observed.

In addition to this general pattern, individual Criollo breeds showed some particular features regarding their relationships with other breeds. For example, the dendrogram based on Nei’s genetic distances, clustered 13 out of the 17 CRI populations with the BIS and CEL breeds from the Iberian group, suggesting that the Iberian influence on Criollos has been mostly through the Celtic group of breeds, which includes BIS and CEL (from the northern Iberian Peninsula), rather than from breeds of the Mediterranean group (from the south). This feature is further supported by the analyses with STRUCTURE, where various Criollo breeds share a common ancestry with the CEL breed, while others share ancestry with BIS and NMA, which are of Celtic-type. This was somewhat unexpected, as most ships sailing to America started their journey in southern Spain and the majority of the first explorers and settlers also came from the Extremadura and Andalusia regions in southern Spain [77]. In this region, the Mediterranean type of pigs prevails, in association with the oak forests where they graze, while Celtic breeds of pigs are found mostly in the northern part of the Iberian peninsula [12]. Therefore, it could be expected that pigs brought on board to be taken to America would be essentially of the Mediterranean type [78]. Our results, however, do not support this idea, and indeed they rather indicate the arrival in America of Celtic-type pigs from the northern Iberian Peninsula, which have left an indelible mark in Criollo pigs.

Our study also uncovered a few other aspects for some particular breeds, including the clear influence of wild boars on the genetic make-up of BOL and BCS, which certainly reflects admixture of these populations with wild boars that have been introduced in the Americas for hunting, at least since the early 19th century [79].

As revealed in the analyses with STRUCTURE, the vast majority of Criollo breeds have received contributions from a large number of ancestral populations, which they share with each other in most cases, even though in a heterogeneous way. However, Criollos generally differ from the other breeds included in our analyses, with the exception of Iberian breeds, whose contribution is often present in various Criollo breeds.

Overall, this study shows that a diversified, but generally common, genetic background underlies the foundations of the gene pool of the majority of the Criollo breeds. Furthermore, all analyses also indicate that, in spite of this common background, Criollo breeds have diverged over time, and they currently differ from each other, such that most of them have their own identity and deserve to be recognized and protected to prevent their extinction, given the endangered status of many of them. The results of our study can be used to establish programs and strategies aimed at the conservation of this genetic pool, to be developed by the appropriate national institutions and breeders, according to the guidelines recommended by FAO [80].

The long history of Criollo pigs in the Americas, starting from a narrow founder population and then expanding throughout the continent and evolving to adapt to extremely different environmental conditions, provides a unique opportunity to investigate the genetic mechanisms underlying adaptation to different challenges, including temperature, humidity, altitude, feed constraints, health challenges, etc. Further studies at the level of the whole genome are warranted to clarify these issues, for which the maintenance and in-depth study of Criollo populations are crucial.

## Conclusions

Our study with a broad representation of American Criollo pig populations and breeds representing their presumed ancestors reveals that Criollo pigs present higher levels of genetic diversity with a distinct identity, and in most cases Criollo breeds remain well differentiated. The influence of Iberian breeds, particularly of the Celtic group, is still present in various Criollo breeds, and a few Criollos reveal genetic contributions from Commercial breeds or from wild pigs. Our results can be instrumental for the recognition of Criollo breeds of pigs, to acknowledge the role that they play in rural development and for establishing the basis of selection and conservation programs aimed at their sustainable use. Furthermore, the long history of adaptation of Criollos to very diverse environmental conditions, after diverging from a common founder population, provides a unique opportunity to investigate genetic mechanisms underlying the ability to cope with various environmental challenges, which is an insight of major importance in a scenario of climate change.

## Supporting information

S1 Fig Representation of the Delta K estimate for different values of K. The Delta K graph indicated a maximum value at K = 29.
(TIF)

S1 Table Populations studied, corresponding acronym, country of origin, sample size (n) and diversity estimates: total number of alleles observed in each breed (tA), mean number of alleles per locus (MNA), allelic richness (R_t_), effective number of alleles (Ae) expected (H_e_) and observed (H_o_) heterozygosities, inbreeding coefficient (*f*) and its significance (* p<0.05; ** p<0.01; *** p<0.001). (DOCX)

S2 Table Effective population size (Ne) and corresponding 95% confidence interval (CI); Wilcoxon sign-rank tests for heterozygosity excess assuming a Two-Phase model (TPM) and an Infinite allele model (IAM); Garza-Williamson index (GW), in 39 pig breeds and 4 wild boar populations. (DOCX)

S3 Table Partitioning of the genetic variability among the different sources of variation by AMOVA, considering population groups. (DOCX)
