# Supplementary figures and images for: On the origins of American Criollo pigs: A common genetic background with a lasting Iberian signature
Source: PLoS One. 2021 May 20;16(5):e0251879. doi: 10.1371/journal.pone.0251879 (PMC8136715; doi:10.1371/journal.pone.0251879)

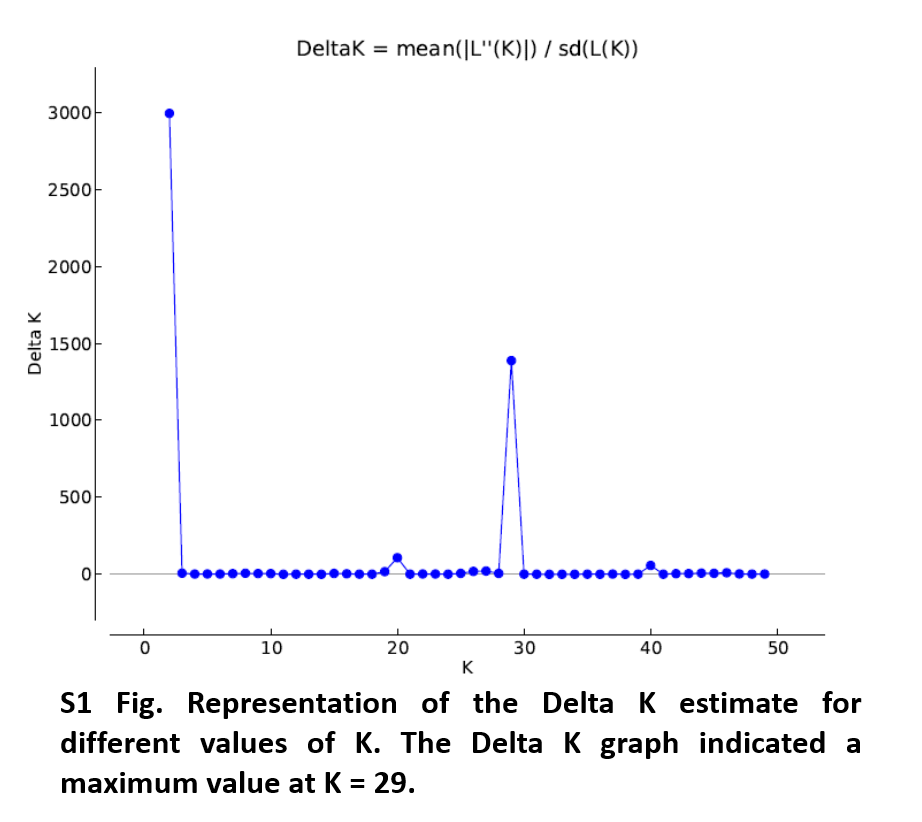

Supplement: S1 Fig — The Delta K graph indicated a maximum value at K = 29. (TIF) [file pone.0251879.s001.tif]
