# Supplementary material for: On the origins of American Criollo pigs: A common genetic background with a lasting Iberian signature
Source: PLoS One. 2021 May 20;16(5):e0251879. doi: 10.1371/journal.pone.0251879 (PMC8136715; doi:10.1371/journal.pone.0251879)
Supplement: S1 Table — (DOCX) [file pone.0251879.s002.docx]

**S1 Table. Populations studied, corresponding acronym, country of origin, sample size (n) and diversity estimates: total number of alleles observed in each breed (tA), mean number of alleles per locus (MNA), allelic richness (R_t_), effective number of alleles (Ae) expected (H_E_) and observed (H_O_) heterozygosities, inbreeding coefficient (F_IS_) and their significance (* *P*<0.05; ** *P*<0.01; *** *P*<0.001) and number of loci in Hardy-Weinberg disequilibrium (LHWEd) with *P*<0.0001.**

| ***Population name*** | | | ***Acronym*** | ***Country*** | ***n*** | ***tA*** | ***MNA (SD)*** | ***Rt (SD)*** | ***Ae (SD)*** | ***H_E_ (SD)*** | ***H_O_ (SD)*** | ***f*** | ***LHWEd*** |
| --- | --- | --- | --- | --- | --- | --- | --- | --- | --- | --- | --- | --- | --- |
| **CRIOLLO** | Mulefoot | | UMF | USA | 36 | 78 | 3.25 (1.07) | 2.49 (0.70) | 1.90 (0.59) | 0.428 (0.039) | 0.411 (0.018) | 0.040 | 2 |
|  | Red Watle Hog | | RWH | USA | 35 | 102 | 4.25 (2.13) | 3.19 (0.81) | 2.14 (0.67) | 0.494 (0.034) | 0.480 (0.018) | 0.028 | 0 |
|  | Guinea Hog | | UGH | USA | 34 | 124 | 5.17 (1.55) | 3.67 (1.11) | 2.65 (1.07) | 0.561 (0.045) | 0.483 (0.019) | 0.141*** | 2 |
|  | Criollo Baja California Sur | | BCS | Mexico | 20 | 130 | 5.42 (2.28) | 4.27 (1.61) | 3.31 (1.56) | 0.628 (0.047) | 0.554 (0.025) | 0.122*** | 0 |
|  | Pelon Mexicano | | MEX | Mexico | 49 | 156 | 6.50 (2.09) | 4.68 (1.53) | 3.49 (1.74) | 0.629 (0.045) | 0.451 (0.015) | 0.285*** | 10 |
|  | Criollo de El Salvador | | SAL | El Salvador | 21 | 151 | 6.29 (2.48) | 4.79 (1.49) | 3.78 (2.08) | 0.667 (0.039) | 0.569 (0.023) | 0.151*** | 1 |
|  | Criollo Cubano | | CUB | Cuba | 50 | 152 | 7.60 (2.16) | 4.91 (1.38) | 3.48 (1.46) | 0.649 (0.043) | 0.647 (0.015) | 0.003 | 1 |
|  | Criollo de Guadalupe | | GUA | Guadalupe | 35 | 171 | 7.13 (2.88) | 4.93 (1.62) | 3.93 (1.85) | 0.689 (0.038) | 0.639 (0.017) | 0.074*** | 0 |
|  | Criollo Venezolano | | VEN | Venezuela | 30 | 152 | 6.33 (2.48) | 4.51 (1.60) | 3.64 (1.98) | 0.645 (0.045) | 0.593 (0.019) | 0.083*** | 2 |
|  | Zungo | | ZUN | Colombia | 33 | 124 | 5.17 (1.88) | 4.02 (1.16) | 2.89 (1.14) | 0.604 (0.037) | 0.577 (0.019) | 0.044* | 0 |
|  | Sanpedreño | | CSP | Colombia | 14 | 121 | 5.04 (2.01) | 4.26 (1.54) | 3.28 (1.79) | 0.634 (0.041) | 0.644 (0.027) | -0.017 | 0 |
|  | Criollo del Pacífico | | CCP | Colombia | 42 | 191 | 7.96 (2.27) | 5.19 (1.08) | 3.85 (1.71) | 0.690 (0.033) | 0.636 (0.016) | 0.080*** | 0 |
|  | Criollo Ecuatoriano | | ECU | Ecuador | 50 | 209 | 8.71 (3.20) | 5.25 (1.42) | 4.20 (2.22) | 0.696 (0.040) | 0.610 (0.014) | 0.125*** | 3 |
|  | Criollo Boliviano | | BOL | Bolivia | 34 | 173 | 7.21 (3.30) | 4.86 (1.69) | 3.84 (2.03) | 0.657 (0.045) | 0.629 (0.017) | 0.044* | 2 |
|  | Pampa Rocha | | UPR | Uruguay | 32 | 122 | 5.08 (2.04) | 4.22 (1.26) | 2.82 (1.84) | 0.581 (0.042) | 0.570 (0.018) | 0.019 | 0 |
|  | Criollo Argentina Wet | | NEW | Argentina | 50 | 197 | 8.21 (2.13) | 5.28 (1.23) | 3.91 (1.70) | 0.698 (0.031) | 0.605 (0.015) | 0.135*** | 4 |
|  | Criollo Argentina Dry | | NED | Argentina | 40 | 163 | 6.79 (3.02) | 4.38 (1.64) | 3.35 (1.62) | 0.626 (0.044) | 0.548 (0.016) | 0.126*** | 2 |
|  | **Criollo** | | **CRI** |  | **605** | **332** | **13.83 (5.00)** | **7.98 (2.09)** | **4.64 (2.87)** | **0.702 (0.036)** | **0.566 (0.004)** | **0.194***** |  |
| **IBERIAN PENINSULA** | Retinto | | RET | Spain | 50 | 131 | 5.46 (2.64) | 3.69 (1.25) | 2.70 (1.13) | 0.547 (0.050) | 0.522 (0.014) | 0.046* | 3 |
|  | Entrepelado | | ENT | Spain | 50 | 133 | 5.54 (2.25) | 3.87 (1.34) | 2.82 (1.16) | 0.564 (0.050) | 0.556 (0.014) | 0.015 | 1 |
|  | Torbiscal | | TOR | Spain | 50 | 116 | 4.83 (2.44) | 3.42 (1.36) | 2.58 (1.34) | 0.529 (0.050) | 0.468 (0.015) | 0.116*** | 3 |
|  | Negro de los Pedroches | | NPE | Spain | 29 | 101 | 4.21 (1.41) | 3.54 (1.12) | 2.58 (1.02) | 0.561(0.040) | 0.554 (0.020) | 0.012 | 1 |
|  | Lampiño | | LAM | Spain | 50 | 140 | 5.83 (2.85) | 4.00 (1.62) | 2.91 (1.40) | 0.575 (0.048) | 0.526 (0.014) | 0.086*** | 2 |
|  | Manchado de Jabugo | | MJA | Spain | 41 | 81 | 3.38 (1.64) | 2.83 (1.23) | 1.96 (0.94) | 0.388 (0.051) | 0.397 (0.016) | -0.021 | 1 |
|  | Chato Murciano | | CHM | Spain | 50 | 108 | 4.50 (2.02) | 3.15 (1.15) | 2.09 (0.70) | 0.460 (0.044) | 0.393 (0.014) | 0.147*** | 1 |
|  | Negro Canario | | NCA | Spain | 50 | 139 | 5.79 (1.84) | 3.78 (1.14) | 2.66 (1.02) | 0.554 (0.046) | 0.488 (0.015) | 0.119*** | 3 |
|  | Negro de Formentera | | NFO | Spain | 20 | 92 | 3.83 (1.58) | 2.99 (1.19) | 1.93 (0.71) | 0.423 (0.045) | 0.349 (0.022) | 0.179*** | 4 |
|  | Negro Mallorquín | | NMA | Spain | 20 | 111 | 4.63 (1.47) | 3.77 (1.21) | 2.51 (1.01) | 0.560 (0.034) | 0.514 (0.023) | 0.084** | 3 |
|  | Euskal Txerria | | ETX | Spain | 50 | 92 | 3.83 (1.95) | 2.70 (1.22) | 1.93 (0.83) | 0.389 (0.052) | 0.390 (0.014) | -0.003 | 0 |
|  | Celta | | CEL | Spain | 27 | 109 | 4.54 (1.79) | 3.68 (1.39) | 3.04 (1.43) | 0.596 (0.047) | 0.496 (0.021) | 0.171*** | 0 |
|  | Alentejano | | ALE | Portugal | 50 | 155 | 6.46 (3.04) | 4.20 (1.57) | 3.26 (1.58) | 0.587 (0.055) | 0.521 (0.015) | 0.113*** | 2 |
|  | Bisaro | | BIS | Portugal | 49 | 134 | 5.58 (2.26) | 4.12 (1.26) | 3.36 (1.56) | 0.632 (0.041) | 0.549 (0.015) | 0.132*** | 4 |
|  | Malhado de Alcobaça | | MAL | Portugal | 36 | 88 | 3.67 (1.09) | 2.91 (0.72) | 2.23 (0.63) | 0.522 (0.031) | 0.514 (0.018) | 0.015 | 0 |
|  | **Iberian** | | **IBE** |  | **622** | **281** | **11.71 (4.72)** | **6.88 (2.22)** | **3.90 (1.81)** | **0.663 (0.043)** | **0.484 (0.004)** | **0.271***** |  |
| **LOCAL BRITISH** | | Berkshire | BSH | UK | 47 | 76 | 3.17 (1.31) | 2.55 (0.96) | 1.76 (0.58) | 0.443 (0.041) | 0.447 (0.015) | -0.009 | 0 |
|  |  | Tamworth | TWR | UK | 41 | 78 | 3.25 (1.07) | 2.72 (0.67) | 2.24 (0.68) | 0.517 (0.034) | 0.519 (0.016) | -0.003 | 0 |
|  |  | Large Black | LBL | UK | 47 | 108 | 4.50 (1.79) | 3.18 (1.17) | 2.45 (0.86) | 0.537 (0.042) | 0.509 (0.015) | 0.053** | 1 |
|  | | **Local British** | **LBR** |  | **135** | **144** | **6.00 (1.98)** | **4.87 (1.47)** | **3.27 (1.29)** | **0.640 (0.033)** | **0.489 (0.009)** | **0.236***** |  |
| **COMMERCIAL** | Duroc | | DUR | USA | 50 | 97 | 4.04 (1.49) | 3.15 (0.86) | 2.61 (1.14) | 0.556 (0.038) | 0.548 (0.014) | 0.013 | 0 |
|  | Pietrain | | PIE | UK | 46 | 125 | 5.21 (1.61) | 3.75 (0.99) | 2.82 (0.93) | 0.599 (0.037) | 0.603 (0.015) | -0.008 | 0 |
|  | Large White | | LWH | UK | 29 | 110 | 4.58 (1.61) | 3.28 (0.94) | 2.58 (0.90) | 0.564 (0.040) | 0.548 (0.019) | 0.029 | 2 |
|  | Landrace | | LDR | UK | 26 | 106 | 4.42 (1.50) | 3.63 (1.13) | 2.70 (0.91) | 0.600 (0.029) | 0.571 (0.020) | 0.050* | 0 |
|  | Large White x Landrace | | LLW | UK | 19 | 107 | 4.46 (1.64) | 3.85 (0.96) | 2.74 (0.86) | 0.613 (0.030) | 0.667 (0.022) | -0.091** | 0 |
|  | **COMMERCIAL** | | **COM** |  | **170** | **200** | **8.33 (3.38)** | **6.04 (1.72)** | **4.08 (1.70)** | **0.708 (0.030)** | **0.581 (0.008)** | **0.180***** |  |
| **MANGALIÇA** | Mangaliça | | **MAN** | Hungary | 25 | 76 | 3.17 (1.27) | 2.74 (1.02) | 1.99 (0.74) | 0.424 (0.050) | 0.347 (0.020) | 0.185*** | 0 |
|  |  | |  |  | **25** | **76** | **3.17 (1.27)** | **3.16 (1.21)** | **1.99 (0.74)** | **0.424 (0.050)** | **0.347 (0.020)** | **0.185***** |  |
| **MEISHAN** | Meishan | | **MSH** | China | **45** | **105** | **4.38 (1.47)** | **3.76 (0.92)** | **2.66 (0.99)** | **0.584 (0.033)** | **0.522 ( 0.016)** | **0.108***** | 2 |
| **WILD BOAR** | Portuguese wild boar | | PWB | Portugal | 39 | 156 | 6.50 (2.27) | 4.22 (1.47) | 3.00 (1.44) | 0.592 (0.046) | 0.561 (0.017) | 0.055* | 1 |
|  | Spanish wild boar | | SWB | Spain | 50 | 151 | 6.29 (3.43) | 4.13 (1.59) | 3.36 (2.03) | 0.600 (0.050) | 0.545 (0.014) | 0.092*** | 2 |
|  | Polish wild boar | | LWB | Poland | 12 | 107 | 4.46 (2.04) | 3.79 (1.72) | 2.96 (1.52) | 0.579 (0.055) | 0.576 (0.029) | 0.006 | 0 |
|  | Italian wild boar | | IWB | Italy | 12 | 73 | 3.04 (1.20) | 2.75 (0.92) | 2.05 (0.81) | 0.457 (0.047) | 0.489 (0.030) | -0.074 | 0 |
|  | **Wild boar** | | **WBO** |  | **113** | **216** | **9.00 (4.18)** | **6.23 (2.53)** | **4.08 (2.69)** | **0.648 (0.047)** | **0.549 (0.010)** | **0.152***** |  |
|  |  | | Total/Average |  | 1715 | 384 | 5.21 (1.43) | 5.87 (1.53) | 2.85 (0.64) | 0.568 (0.082) | 0.529 (0.079) | 0.067 |  |
