# Supplementary material for: On the origins of American Criollo pigs: A common genetic background with a lasting Iberian signature
Source: PLoS One. 2021 May 20;16(5):e0251879. doi: 10.1371/journal.pone.0251879 (PMC8136715; doi:10.1371/journal.pone.0251879)
Supplement: S2 Table — (DOCX) [file pone.0251879.s003.docx]

**S2 Table. Partitioning of the genetic variability among the different sources of variation by AMOVA, considering population groups**

| **Source of variation** | **Degrees of freedom** | **Sum of squares** | **Variance component** | **Percentage of variation** |
| --- | --- | --- | --- | --- |
| Among groups^1^ | 6 | 993.0 | 0.227 | 4.44 |
| Among populations within groups | 39 | 2666.8 | 0.869 | 17.05 |
| Among individuals within populations | 1669 | 6926.9 | 0.146 | 2.86 |
| Within individuals | 1715 | 6617.0 | 3.858 | 75.64 |

^1^ See Table 1 for specification of breeds/populations belonging to each group.
