# Supplementary material for: On the origins of American Criollo pigs: A common genetic background with a lasting Iberian signature
Source: PLoS One. 2021 May 20;16(5):e0251879. doi: 10.1371/journal.pone.0251879 (PMC8136715; doi:10.1371/journal.pone.0251879)
Supplement: S3 Table — (DOCX) [file pone.0251879.s004.docx]

**S3 Table. Effective population size (Ne) and 95% confidence interval; Wilcoxon sign-rank tests for heterozygosity excess and Garza-Williamson index (G-W) in 39 pig breeds and 4 wild boar populations, TPM: Two-Phase model; IAM: Infinite allele model.**

|  |  | **Ne** | **95% CI** | **TPM-Hexcess** | **IAM-Hexcess** | **G-W** |
| --- | --- | --- | --- | --- | --- | --- |
| **CRIOLLOS** | Mulefoot | 29.6 | 20.6-47.2 | 0.18 | 0.01 | 0.57 |
|  | Red Watle Hog | 12.7 | 10.4-15.6 | 0.55 | 0.05 | 0.54 |
|  | Guinea Hogh | **7.7** | 6.6-9 | 0.81 | 0.11 | 0.63 |
|  | Criollo Baja California Sur | 60.9 | 34.9-186.9 | 0.27 | 0.02 | 0.59 |
|  | Pelon Mexicano | 10.1 | 9.4-11 | 0.37 | 0.01 | 0.64 |
|  | Criollo de El Salvador | 248.1 | Infinite | 0.66 | 0.01 | 0.66 |
|  | Criollo Cubano | 64.7 | 53.9-79.8 | 0.87 | 0.13 | 0.74 |
|  | Criollo de Guadalupe | 16.7 | 15-18.6 | 0.33 | 0.00 | 0.66 |
|  | Criollo Venezolano | 24.6 | 20.6-29.8 | 0.45 | 0.04 | 0.68 |
|  | Zungo | 114.1 | 61.1-547.2 | 0.44 | 0.00 | 0.66 |
|  | Sanpedreño | **7.8** | 6.0-10.0 | 0.29 | 0.04 | 0.56 |
|  | Criollo del Pacífico | 149.8 | 108.2-236.2 | 0.89 | 0.04 | 0.67 |
|  | Criollo Ecuatoriano | 65.5 | 57.7-75.2 | 0.75 | 0.01 | 0.76 |
|  | Criollo Boliviano | 20.3 | 18-23.1 | 0.74 | 0.04 | 0.68 |
|  | Pampa Rocha | 35.9 | 28.3-47.5 | 0.48 | 0.00 | 0.65 |
|  | Criollo Argentina Wet | 33.3 | 30.1-37 | 0.88 | 0.01 | 0.74 |
|  | Criollo Argentina Dry | 20.7 | 18.7-23.1 | 0.74 | 0.05 | 0.71 |
| **IBERIAN PENINSULA** | Retinto | 20.7 | 18.4-23.3 | 0.65 | 0.09 | 0.6 |
|  | Entrepelado | 38.6 | 21.6-88.3 | 0.41 | 0.08 | 0.63 |
|  | Torbiscal | 32 | 27.3-38.0 | 0.22 | 0.00 | 0.62 |
|  | Negro de los Pedroches | 4.6 | 2.6-8.7 | 0.09 | 0.00 | 0.58 |
|  | Lampiño | 16.9 | 15.3-18.7 | 0.57 | 0.00 | 0.65 |
|  | Manchado de Jabugo | 6.7 | 5-8.2 | 0.41 | 0.01 | 0.62 |
|  | Chato Murciano | 4.7 | 3.9-6 | 0.92 | 0.13 | 0.63 |
|  | Negro Canario | 15.8 | 14.2-17.5 | 0.92 | 0.25 | 0.59 |
|  | Negro de Formentera | **2.1** | **1.9-2.4** | 0.98 | 0.85 | 0.59 |
|  | Negro Mallorquín | 23 | 17.4-32 | 0.85 | 0.17 | 0.56 |
|  | Celta | **63.9** | **38.4-158.4** | **0.02** | 0.00 | 0.54 |
|  | Euskal Txerria | 23 | 18.9-20.2 | 0.84 | 0.28 | 0.55 |
|  | Alentejano | **54.9** | 46.9-59.3 | 0.79 | 0.05 | 0.68 |
|  | Bisaro | 9.5 | 8.6-10.6 | **0.01** | 0.00 | 0.61 |
|  | Malhado | 11.6 | 9.4-14.3 | **0.02** | 0.00 | 0.56 |
| **BRITISH** | Berkshire | 38.5 | 28.2-55.8 | **0.01** | 0.00 | 0.5 |
|  | Tamworth | 28.9 | 21.7-40.3 | **0.00** | 0.00 | 0.55 |
|  | Large Black | 61 | 46.6-84.7 | 0.25 | 0.00 | 0.5 |
| **COMMERCIAL** | Duroc | 76.5 | 56.3-113.3 | **0.00** | 0.00 | 0.57 |
|  | Pietrain | 32.1 | 27.6-37.7 | 0.23 | 0.00 | 0.57 |
|  | Large White | 30 | 22.9-41.3 | 0.25 | 0.01 | 0.58 |
|  | Landrace | 38.2 | 27.7-57.9 | **0.02** | 0.00 | 0.56 |
|  | Large white x Landrace | 21.4 | 16.1-30 | 0.09 | 0.00 | 0.56 |
| **MANGALIÇA** | Mangalica | 2.6 | 2.3-3 | 0.12 | 0.00 | 0.55 |
| **MEISHAN** | Meishan | 15.1 | 13.1-17.4 | 0.00 | 0.00 | 0.53 |
| **WILD BOAR** | Portuguese wild boar | 78.5 | 56.6-122.8 | 0.99 | 0.42 | 0.68 |
|  | Spanish wild boar | 26.1 | 23.5-29 | 0.20 | 0.00 | 0.7 |
|  | Polish wild boar | 13.4 | 9.4-20.3 | 0.31 | 0.02 | 0.64 |
|  | Italian wild boar | 43.7 | Infinite | 0.23 | 0.03 | 0.55 |
